# Supplementary material for: Patterns of Intron Gain and Loss in Fungi
Source: PLoS Biol. 2004 Nov 30;2(12):e422. doi: 10.1371/journal.pbio.0020422 (PMC532390; doi:10.1371/journal.pbio.0020422)
Supplement: Table S1 — Also available at http://genes.mit.edu/NielsenEtAl/. (4.3 MB ZIP). [file pbio.0020422.st001.zip › NielsenEtAl/html/1438.html]

AN3098.1.NCU03387.1.MG02418.1.FG10903.1


```
 CLUSTAL W (1.82) Multiple Sequence Alignments - Introns Inserted


Sequence 1: NCU03387.1	853 aa
Sequence 2: MG02418.1	835 aa
Sequence 3: FG10903.1	822 aa
Sequence 4: AN3098.1	775 aa
Alignment Length: 874 aa
Number Identitical Residues: 286 aa
Alignment Score (without introns) 15761


MG02418.1 	------MDMRNNLFGQGPRGSSG--GLPGRQVQRPPGG---GAPPPG~RYDSPQG-----
NCU03387.1	------MDIRGNLFGKKPQTPQGGAGLPGRPVQQAAGGGYGGGATPT~NYGSPGGRAPSQ
FG10903.1 	MSRTPFSGFMGGSRGQQPPPPQQQQGYGGPRYNDPRQQ----QQPPQ~NYQSYPG-----
AN3098.1  	------MFNRNNYSNPFGSNPPQRDGYSRPPQGYPPQHG---APDSG2GYGTPPP-----
          	          ..  .     .    *        .   .      .   * :        

MG02418.1 	----GYGGGNYAPPPSQQPRQPSG---YGNPPPADNYG-APSGRRVRLRLAKVEDK-TLQ
NCU03387.1	PQYSGSGAGGYGGAPSEKSYGGGGGGGYGGQPAPGGYPSQPAGRGVPLRLAKVDDK-TLQ
FG10903.1 	----GSGGGGYAEKPSR-----------------------SGGRQVSLRVEKVTDK-SLQ
AN3098.1  	----RRPAPGARPPPQAP----------------------MGGRPSEGGVWALTPKESPN
          	       . .    *.                         .**     :  :  *.: :

MG02418.1 	MNYIFTNI2CAVSPSDFPSSRDG-DHYIRLTGPQLRGDYVVMARPTPGFPPGCISLSDPQ
NCU03387.1	SLYIFGNI2CAVSPNDFPPNHDGTDLYIRLTGRQLRGDYVVTARPTPGFRPGHISLSDPQ
FG10903.1 	SRLIYGNL2CAVSPDDFPPNRDGSDLYILLQAGQPMGEYVVTAKPVPGFPQGCISLSDPQ
AN3098.1  	KECQFGNL2VALSAQDFPRAQFGYEDILIIVN----GLYVFSARILDEFPPGYIGLSSIQ
          	    : *:  *:*..***  : * :  : :      * **. *:    *  * *.**. *

MG02418.1 	RTWLQVGLMDELEGELYDPLG-GGTPYLGAIDVEVGFASKAKFAEEAYDQDELQRMMVS~
NCU03387.1	RTWCGVGMMDELMGEVYDPFARGGDAYIGSMDVEIGFASTNRIVDTPYDQDELAKVFLK~
FG10903.1 	RSWAGITMRDVFQGEIYDPFAAGGKAYLGSVNLEIGFASPNKKTDVPYDEDELSKMFIE~
AN3098.1  	RPWARAGFRDSLDVRIYDPFRQGGEAYLGSADMEVKFAGKLR-PDTLYDQDELLNSVIK0
          	*.*    : * :  .:***:  ** .*:*: ::*: **.  :  :  **:*** . .:. 

MG02418.1 	TFANQMFAPGQRILMDVRSIPLAIQIKTVTLVDLSMSEKPAGDAAMTSSDPNKRGVLTNQ
NCU03387.1	TFQNQVFAPGQRFLLDVKNIPLAVMVKTVTLVDLTMLG---SGEAPTRSDPGARGILTNQ
FG10903.1 	TYQNQVLAPGQRILMDHRNIPLLIVVKTVSLTDLAMTSD--DSSKKAQREPHARGILTTQ
AN3098.1  	NFESQIFAPGQPVLMDHHGVPLQLTVKTILRVSLTSEK---DTSKVPETEPTARGILTKH
          	.: .*::**** .*:* :.:** : :**:  ..*:      .    .  :*  **:**.:

MG02418.1 	TGVTFFKDPKTSIKLKASSRKAASNAIVSPDFKFEDMGIGGLDNEFATIFRRAFASRIFP
NCU03387.1	ASIGFYKDARSPIKLKGSNKRPAANAIIAPDFKFENMGIGGLDTEFSTIFRRAFASRIFP
FG10903.1 	SQVVFHRDAKGDFNLKPSMHKPNSNAILAPDFKFEDMGIGGLDDEFATIFRRAFASRIFP
AN3098.1  	TLINFFKDPQSEIQIKPAKNRPAANAIIQPDFNTEKMGIGGLDSEFHTIFRRAFASRIFP
          	: : *.:*.:  :::* : .:. :***: ***: *.******* ** *************

MG02418.1 	PELIEKLGIQHVKGILLFGPPGTGKTLIARQIGKMLNAREPKIINGPEVLNKYVGQSEEN
NCU03387.1	PGLIEKLGIMHVKGMLLYGPPGTGKTLIARQIGKMLNAREPKVINGPEVLNKYVGQSEEN
FG10903.1 	PGLIAKMGITHVKGLLLYGPPGTGKTLIARQIGKMLNANPPKVINGPEVLNKYVGQSEEN
AN3098.1  	PDIVQKLGIQHVKGILLFGPPGTGKTLLARQIGKMLNAREPKIINGP-------------
          	* :: *:** ****:**:*********:**********. **:****             

MG02418.1 	IRKMFADAEKEYAEKGDESGLHIIIFDELDAVCKQ~RGSGSGGGTGVGDSVVNQLLSKLD
NCU03387.1	IRKLFADAEKEYKEKGDESGLHIIIFDELDAVCKQ~RGSGAGGGTGVGDSVVNQLLSKLD
FG10903.1 	IRKLFADAEKEYKEKGDESSLHIIIFDELDAVCKQ~RGSGSGGGTGVGDSVVNQLLAKLD
AN3098.1  	----------------------------------E0RGSGAGGGTGVGDSVVNQLLSKMD
          	                                  : ****:***************:*:*

MG02418.1 	GVNQLNNILLIGMTNRKDMIDDALLRPGRLEVQVEISLPDEPGREKILQIHTTKMVKNNV
NCU03387.1	GVDQLNNILLIGMTNRKDMIDDALLRPGRLEVQIEISLPDEHGRGQILKIHTSKMKENKV
FG10903.1 	GVDQLNNILLIGMTNRKDMIDDALLRPGRLEVHLEISLPDEAGRLDILKIHTAKMASNGL
AN3098.1  	GVDQLNNILLIGMTNRKDMIDDALLRPGRLEVHVEISLPDEAGRAQILGIHTQNMRQSDL
          	**:*****************************::******* ** .** *** :* .. :

MG02418.1 	IESDVDLRELAAMTKNFSGAEISGLVKAATSFAFNRHTKVGTMAGVSDNVAEMKVNRDDF
NCU03387.1	MADDVDLAELATLTKNFSGAELSGLVKSATSYAFSRHIKVGTMAGVSEDVANMKVNRQDF
FG10903.1 	LDSSIDLDELAGMTKNFSGAEISGLVKAAASCAFSRHTEVGQLAAVKQDVASMNVKREDF
AN3098.1  	MDPSVNLSELATLTKNYSGAEIAGLVKAATSFAFNRHIDSGKTVRVKDDAAEMKVNHSDF
          	:  .::* *** :***:****::****:*:* **.** . *  . *.::.*.*:*::.**

MG02418.1 	LHALGEVKPAFGMDEDKLGAVIRHGIIHFSPAIDSILRDGMINVDAVRDLEQLNLLSVLL
NCU03387.1	LNALDEVKPAFGADESELEKHLAYGVIHYSKSITSILEDGLLYVNNVRKLERLKHMTVLL
FG10903.1 	MVALTEVRPAYGVSEAELMEAIRLGIYPYAPHIDMNIQQMMRVVGMVKEDPNKFSTSVLF
AN3098.1  	IHALDEIQPAFGVSEDEIKRCIEHGIINYSDKIDNVLQEGEALARGLGRPEQTTLWSVLL
          	: ** *::**:* .* ::   :  *:  ::  *   :.:    .  :    .    :**:

MG02418.1 	HGPEGSGKTAMAAHIAIQSGFPFVKTITAEMLVGCRDEVGKKDMIHKVFTDAYKSPLSLL
NCU03387.1	HGPSASGKTALAAHIAMKSDFPFIKFITPTDMVGFRDEVAKKDYMHKIFTDAYKSPLSLL
FG10903.1 	HGPQGSGKTALAAHIAMQSGFPFVKMVTPSDLVGYRDDFAKKDYVHKAFTDAYKSPASIL
AN3098.1  	NGPPGSGKTALAAQIALDSGAPFIKMVCPEDVAGY-NEAAKIQHILRVFNDAYKSQTSVV
          	:** .*****:**:**:.*. **:* : .  :.*  :: .* : : : *.*****  *::

MG02418.1 	ILDDIEEMIEWNPIGPRMSNMMVQVLRTLMKALPPK0GHRLLILATTSVRSVLQQLDMMK
NCU03387.1	IIDNIERIIEWNPVGPRLSNTILQALVTLLVTPPPK~GHRLLILATTSQRTVMEQLDVTE
FG10903.1 	ILDDFERLIGWNPIGPRFSNTMLEALTTLIVSRPPK~GHRLLVFVTTSKASVLKMLEIDN
AN3098.1  	VVDDIETLIDYVSVGPRFSNSVLQTLKVLFKKRPPK~NRRLLILATTSERALMKELNIYN
          	::*::* :* : .:***:** :::.* .*:   *** .:***::.***  :::: *:: :

MG02418.1 	AFNAEIAIPAIKNLQELSRVLGESAMFESQ--DLNVIVNTIGNITGTD----KVNVGVKT
NCU03387.1	SFDYQIAVPAVNNLQELGAVLNEVGAFGGDQAAVSEVLNTLREYTGSD----SVGVGVRT
FG10903.1 	DFAKKVAVPGVSNLRELAAVLQESPEFEHD---PNAAVNEIGRQTGSD----SVGVGIKT
AN3098.1  	SFNSDIDIPNVTSHEELRHVMEKSEVFASEQ--VAEALERIDPLKGETPYSMTFGVGIKK
          	 *  .: :* :.. .**  *: :   *  :.      :: :   .*  . : ...**::.

MG02418.1 	VLTLASTARKAPD--PVDWFSEEVAKAINNNTPQY----
NCU03387.1	ILTIAESADTSPD--PKGWFVEKMAGLIARYNVR-----
FG10903.1 	ILDCIFESKRDPNGQVIEAFVELVVGKIQELRM------
AN3098.1  	VFDGIELAKKTPD-QLVNQFVRFINSAVQEGGLTTRRAV
          	::     :   *: .    * . :   : .       :
```
